# Neuroendocrine disturbances in women with functional hypothalamic amenorrhea: an update and future directions

**DOI:** 10.1007/s12020-023-03619-w

**Published:** 2023-12-07

**Authors:** Błażej Męczekalski, Olga Niwczyk, Christian Battipaglia, Libera Troia, Anna Kostrzak, Gregory Bala, Marzena Maciejewska-Jeske, Alessandro D. Genazzani, Stefano Luisi

**Affiliations:** 1 Department of Gynecological Endocrinology, Poznan University of Medical Sciences, Poznan, Poland; 2 Gynecological Endocrinology Center, Department of Obstetrics and Gynecology, University of Modena and Reggio Emilia, Modena, Italy; 3 Department of Gynecology and Obstetrics, Maggiore della Carità Hospital, University of Eastern Piedmont, Novara, Italy; 4 UCD School of Medicine University College Dublin, D04 V1W8 Dublin, Ireland; 5 Department of Molecular and Developmental Medicine, University of Siena, Siena, Italy

**Keywords:** Hypothalamic Amenorrhea, Stress, Menstrual Disorders, Weight Loss, Exercise

## Abstract

Functional hypothalamic amenorrhea (FHA) is one of the most common causes of both primary and secondary amenorrhea in women of reproductive age. It is characterized by chronic anovulation and the absence of menses that appear as a result of stressors such as eating disorders, excessive exercise, or psychological distress. FHA is presumed to be a functional disruption in the pulsatile secretion of hypothalamic gonadotropin-releasing hormone, which in turn impairs the release of gonadotropin. Hypoestrogenism is observed due to the absence of ovarian follicle recruitment. Numerous neurotransmitters have been identified which play an important role in the regulation of the hypothalamic-pituitary-ovarian axis and of which the impairment would contribute to developing FHA. In this review we summarize the most recent advances in the identification of contributing neuroendocrine disturbances and relevant contributors to the development of FHA.

## Introduction

Functional hypothalamic amenorrhea (FHA) is a common cause of both primary and secondary amenorrhea among women of reproductive age. It is characterized by chronic anovulation and the absence of menstruation due to inadequate secretion of hypothalamic gonadotropin-releasing hormone (GnRH), which subsequently impairs the release of pituitary gonadotropin and gonadal steroids. Factors such as stress, low weight, and excessive energy expenditure or physical exercise are associated with anovulation in FHA [1]. The prolonged deficiency of estrogen resulting from amenorrhea in reproductive-aged women can have long-term health implications, including bone loss, increased fracture risk, impaired fertility, dyslipidemia, endothelial dysfunction, and an elevated risk of cardiovascular disease [2–4]. Furthermore, women experiencing extended periods of amenorrhea, spanning several months or years, have reported higher rates of sexual dysfunction, depression, and anxiety compared to eumenorrheic women [5]. A comprehensive clinical evaluation is crucial for patients with FHA to identify the underlying causal factors and tailor appropriate interventions. Since stressors can vary significantly among individuals while inducing similar amenorrheic condition, treatment options should be personalized. It is essential to address nutritional and psychosocial stress to restore spontaneous pulsatile release of GnRH and subsequently restore the normal functioning of the hypothalamic-pituitary-ovarian (HPO) axis [6].

## Epidemiology

FHA is the most common cause of primary and secondary amenorrhea in adolescent girls [7]. After pregnancy, FHA and polycystic ovarian syndrome (PCOS) are the most common causes of secondary amenorrhea [9, 10] with FHA being responsible for approximately 25–35% of all cases of secondary amenorrhea [8]. Although many stressors will often compound and lead a patient to develop FHA, caloric restriction is the most prevalent. It is typically accompanied by decreased body fat and circulating leptin levels, increased cortisol and ghrelin levels and a reduction of kisspeptin as well [4, 11–13]. In addition to the well documented contributory lifestyle factors, it has been suggested that the increased susceptibility of some individuals to the effect of stressors on the HPO axis may have a genetic basis [14–16]. Rare or polymorphic variants in genes controlling the development and/or function of GnRH neurons have been recognized in both idiopathic HH and FHA women. Genes which are associated with congenital hypogonadotropic hypogonadism include : the Fibroblast Growth Factor receptor 1 gene (FGFR), prokineticin receptor 2 gene (PROKR2), GNRH receptor gene (GNRHR) and the Kallmann syndrome 1(KAL1) sequence gene (also known as ANOS-1) [17]. Epigenetic changes have been identified which act on pathways of the HPO axis and therefore may be participatory in FHA and may confer on a person a predisposition for anovulation [18].

In a European population study, the prevalence of eating disorders among adolescent and young adult women was reported to be between 0.2 and 4% [19]. Excessive exercise and/or inadequate caloric intake amounting to a relative energy deficiency at the critical time of early development has been shown to delay menarche [4, 7]. Although amenorrhea is no longer a required diagnostic criterion for anorexia nervosa per the Diagnostic and Statistical Manual of Mental Disorders, Fifth Edition (DSM-5) [20], FHA can have a very similar presentation and significant overlap exists between the two conditions. The female athlete triad as a manifestation of FHA imbricates both populations and its etiology. Ultimately, these two conditions are very similar with comparable long-term risks and treatment [21, 22].

## Ethiopathogenesis of FHA

In patients with FHA, the primary cause of anovulation is a functional reduction in GnRH drive and secretion of luteinizing hormone (LH) [23–26]. The reduction in GnRH drive results in impairment of pulsatile LH secretion while follicle-stimulating hormone (FSH) levels become insufficient to maintain full folliculogenesis and ovulatory function [4, 27].

Stressors, regardless of type, activate the hypothalamic–pituitary–adrenal (HPA) axis and autonomic nervous system. This results in a constellation of neuroendocrine, metabolic, and hormonal alterations which ultimately impair pulsatile GnRH release [28]. Changes in thyroid hormone levels is commonly observed, as a consequence of HPA axis activation. Patients with FHA tend to have low thyrotropin-releasing hormone, normal/low TSH, and decreased triiodothyronine (T3) and thyroxine T4. This pattern indicates increased negative feedback of thyroid hormones at the hypothalamus and reduced thyroid responsivity to TSH, as a way to conserve and divert energy expenditure [28–31].

HPA axis activation also increases secretion of corticotrophin-releasing factor (CRH) and finally the endogenous opioid peptide, endorphin and cortisol. The neurotransmitter ƴ-aminobutyric acid (GABA) has also been linked to suppression of GnRH [26, 30]. Changes in thyroid hormone levels are commonly observed. Patients with FHA tend to have lower total triiodothyronine (T3), classically defined as “low T3 syndrome” and total thyroxine (T4) concentrations compared to eumenorrheic controls [31–33]. Decreased leptin [25, 34–36], increased fasting peptide YY [33, 34], and increased fasting ghrelin are all observed in these patients [35, 36]. Kisspeptins are a group of polypeptides that play a key role in the regulation of the reproductive axis by influencing GnRH release. Kisspeptin neurons are found in the hypothalamus, basal ganglia, and periventricular nucleus [37, 38]. Kisspeptin is believed to closely influence the negative and positive feedback of estrogen as a positive correlation has been observed between kisspeptin and LH secretory pulses [18, 39]. The impact of prolonged periods of hypogonadism and changes in serum cortisol, leptin, and peptide YY (PYY) may have on neurocognitive status, emotion, and mood is of particular concern. Such changes pose additional challenges for patients at an age when emotional lability is already strong [6, 40].

## Diagnosis

FHA is a form of chronic anovulation, without any identifiable organic cause that could disrupt the normal frequency of GnRH pulses [13]. The term “functional” implies that restoring ovulatory ovarian function is possible through the correction or improvement of underlying behavioral factors causing the condition [19, 28].

The diagnosis of FHA can be established when amenorrhea occurs alongside low or low-normal LH, normal FSH concentrations, and reduced levels of estradiol (E2) and progesterone. The acute gonadotropin response to GnRH stimulation remains intact [28].

However, specific cutoff values for gonadotropins and estradiol levels, as well as definitive biomarkers or diagnostic tools for FHA diagnosis, have not been determined. The pattern of hormone levels holds greater importance than absolute values. E2 measurements are often of limited value since they reflect a single time point, and a single E2 value alone cannot confirm a diagnosis of FHA. Clinicians should adhere to the Endocrine Society guidelines to assure assay validity and reliability when assessing E2 levels [28].

Amenorrhea persisting for six months or longer is typically observed in adolesceents and young women with FHA. In adolescents, diagnosing FHA poses a challenge due to the immaturity of the HPO axis during the early postmenarchal years. Menstrual status can vary from subclinical menstrual dysfunction to outright oligo-amenorrhea. However, even in the early postmenarchal years, more than 90 percent of reported menstrual cycles in adolescents do not exceed 45 days, making irregular or absent menses a cause for concern [41, 42]. A clinical history must be gathered and particular attention devoted to alterations of the menstrual cycle, time and type of amenorrhea, psychogenic stressors, dieting, exercise patterns, and weight loss [4, 14, 42]. Patterns of disordered eating can include avoidance of certain foods (typically foods high in fat, sugar, and calories), restricting and/or purging (self-induced vomiting, laxative use, or compensatory physical activity). A comprehensive list of medications should be obtained (including non-prescription supplements or additives), and previous or current treatments with chemotherapy or radiation should be noted. Medications such as antidepressants, antipsychotics, and hormonal contraception are known to alter menstruation. Additionally, the chronic use of illicit or controlled substances, such as cocaine and opioids, often accompanies a state of stress and malnutrition [28]. Obtaining a sexual history is crucial, including information on contraceptive use. Furthermore, a thorough family history, including menstrual history of the patient’s biological mother, holds significance [1].

## Clinical presentation

A comprehensive clinical examination, which includes both external and bimanual gynecological examination, is essential as part of the diagnostic work-up, allowing for the evaluation of a wide range of potential differential diagnosis [28]. The physical exam should begin with a general inspection of the patient’s well-being. Biometric data such as the patient’s height and weight should be measured and plotted using an appropriate growth chart and BMI (kg/m2) should be calculated. Vital signs should include blood pressure and heart rate. Measuring body fat percentage using a bioelectrical impedance protocol can help stratify women who exercise intensely or maintain a restrictive diet and who may have an abnormally low body fat percentage despite a normal BMI (a state which is itself associated with ovulatory dysfunction and amenorrhea) [43, 44]. Complete Tanner staging should be assessed to document pubertal development [45]. The presence of signs indicating androgen excess (e.g. acne, hirsutism, male pattern alopecia, clitoromegaly) and hyperinsulinism (such as acanthosis nigricans and skin tags) should raise concerns about PCOS or other potential causes of androgen excess, including nonclassic CAH and virilizing ovarian and adrenal tumors [28]. Additionally, signs of galactorrhea and thyromegaly should be also assessed [28]. A pelvic ultrasound can be helpful in identifying the presence of a uterus and ovaries, and to rule out an adnexal mass. In cases where a Müllerian anomaly is suspected, magnetic resonance imaging (MRI) of the pelvis, or a further 3D transvaginal ultrasound may be indicated to better characterize the specific anomaly [46]. Head imaging using computed tomography (CT) or MRI is not typically required unless galactorrhea is observed in an adolescent patient (+/− hyperprolactinemia), or headaches and visual field changes suggest a possible intracranial lesion [4, 47]. The risk of osteopenia and osteoporosis associated with hypoestrogenism is well documented. Women who experience amenorrhea for six months or more, and particularly those with a history of severe nutritional deficiency, other energy deficit states, and/or skeletal fragility, should undergo baseline assessment of their bone mineral density (BMD) [28, 38]. It is recommended to measure BMD using dual-energy X-ray absorptiometry (DEXA/DXA) scans and lateral spine radiographs to detect asymptomatic vertebral fractures [38–53].

### Hormonal presentation

When evaluating a patient with amenorrhea, it is crucial to include a beta subunit of human chorionic gonadotropin (β-HCG) test in the initial blood work-up, regardless of their disclosed sexual history, to exclude the possibility of pregnancy. Additionally, as part of the initial endocrine evaluation, it is recommended to routinely measure serum concentrations of FSH, LH, estradiol, Anti-Mullerian hormone (AMH), prolactin, thyroid stimulating hormone (TSH), and free T4 [32]. If physical examination reveals signs of hyperandrogenism a comprehensive androgen panel should be ordered including total testosterone, dehydroepiandrosterone sulfate (DHEA-S) and 17-hydroxyprogesterone, preferably collected in the early morning, if there is clinical suspicion of late-onset Congenital Adrenal Hyperplasia (CAH) [4]. To assess for chronic estrogen exposure and to ensure outflow tract integrity, a progestin challenge can be performed to induce withdrawal bleeding [23]. In cases where necessary, additional assessment of serum and urine cortisol may be warranted based on presenting features. The increased secretion of CRH results in increased secretion of adrenocorticotrophin (ACTH) from the pituitary and cortisol from the adrenal glands. These phenomena are associated with a diminished GnRH drive. Moreover, patients with FHA often experience disruptions in the hypothalamic–pituitary–thyroid axis, commonly presenting as “low FT3 syndrome” in the presence of significant caloric deficit [3]. If the patient’s history raises concerns for inflammatory conditions such as inflammatory bowel disease or celiac disease which can manifest as hypogonadism, additional laboratory testing should be considered. This may include a complete blood cell count, chemistry panel, liver panel, erythrocyte sedimentation rate, and/or C-reactive protein. The necessity to add a celiac panel should be considered as well [33]. An elevated random or fasting glucose level should prompt assessment of hemoglobin A1C. Elevated sedimentation rate and/or C-reactive protein levels suggest the presence of chronic inflammatory conditions. Studies have demonstrated that liver function tests are often altered in adolescent and young women with severe energy restrictions [28].

## Kisspeptin neurons as a primary driver of GnRH control

Neuroendocrine control over regular menstrual cycles is regulated by the secretion of gonadotropin releasing hormone from the arcuate nucleus of the hypothalamus, this stimulates the pulsatile release of luteinizing hormone and follicle stimulating hormone. FSH and LH both stimulate follicular growth and development in the ovary leading to the production of estrogen and progesterone [54].

The hypothalamic nuclei release GnRH in an episodic manner. This pulsatile secretion is essential for optimal gonadotropin release and its cadence is maintained by the stimulating or inhibiting action of neurotransmitters and/or neuromodulators [55]. Both psychological and metabolic stressors can induce hormonal changes which impair the secretion of GnRH at the hypothalamic level. This disruption results in abnormal pituitary production of FSH and LH [55, 56].

The pulse frequency itself has a differential effect on LH and FSH secretion, with low-frequency pulses of GnRH preferentially stimulating FSH secretion while high-frequency GnRH secretion stimulates production and release of LH [57, 58].

**Fig. 1:**
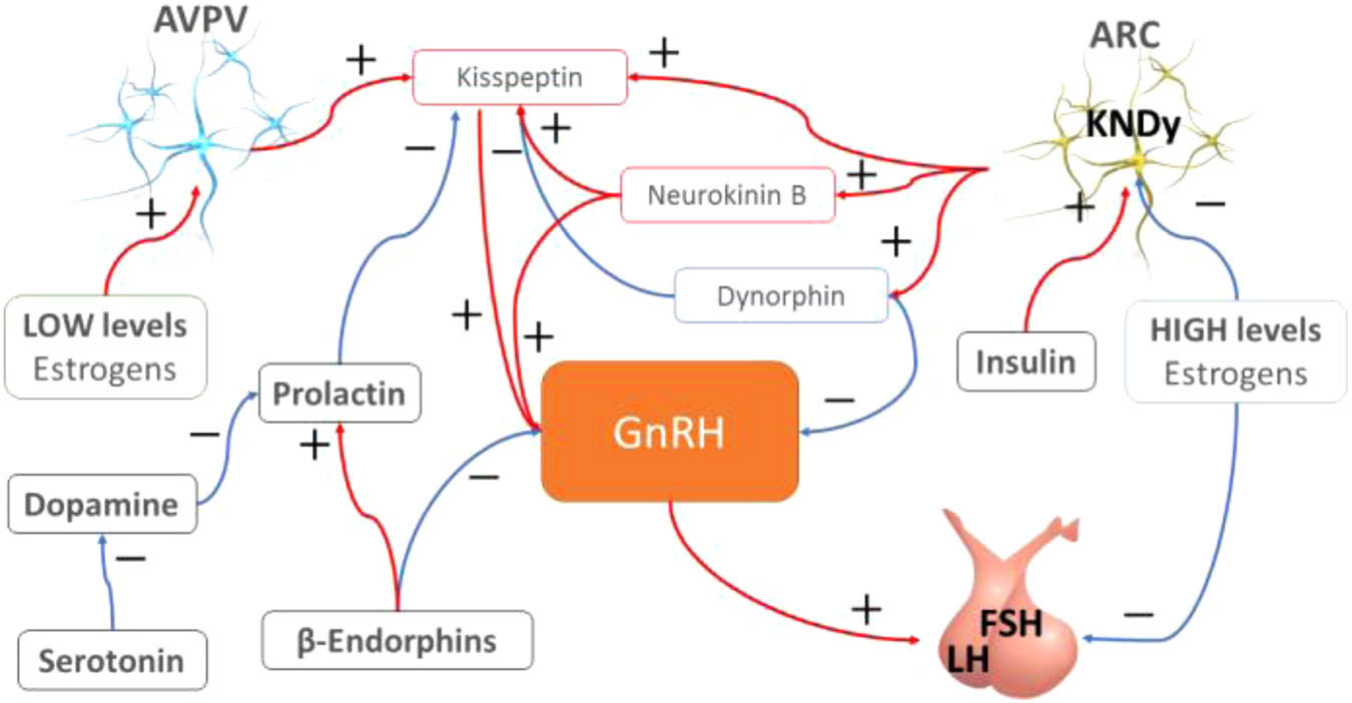
The complex interconnections that link Kisspeptin secreting neurons and GnRH secretion to drive gonadotropin discharge from gonadotropic cells as studied in rodents

GnRH pulsatility and subsequent FSH and LH discharge is regulated by kisspeptin/neurokinin B/dynorphin (KNDy) secreting neurons located in the arcuate nucleus (ARC) [59] (Fig. 1).

Kisspeptin(Kp) is also produced by a second group of neurons located in the rostral periventricular area of the third ventricle corresponding to the anteroventral periventricular nucleus (AVPV) of the hypothalamus. These neurons were demonstrated in a rodent model to be devoid of neurokinin-B or dynorphin production and mediate positive feedback from estrogens [60].

Several studies conducted on autopsy samples have shown that in humans, kisspeptin neurons are primarily located in the infundibular nucleus, which is the homolog of the ARC in rodents, as well as in the rostral preoptic area (POA) [61].

Kp is a hormone encoded by the KISS1 gene located on chromosome 1q32. It plays a crucial role in stimulating, releasing, and amplifying GnRH pulses [62, 63]. KNDy neurons express receptors for Neurokinin B (NKB), dynorphin (DYN), as well as estradiol (ERα) and progesterone (PR) [64].

Through these receptors, NKB stimulates the secretion of kisspeptin from KNDy neurons. By the same pathway, dynorphin inhibits kisspeptin and suppresses GnRH pulse frequency [65].

GnRh secretion occurs in two distinct modes: surge and the pulsatile modes [66].

Pulsatile secretion of GnRH promotes follicle maturation and triggers the release of gonadotropins during the menstrual cycle, ultimately leading to sex hormone production. Importantly, Kp neurons in the ARC are tonically suppressed by estrogen signals. As a result, elevated levels of sex steroid hormones inhibit or modulate kisspeptin secretion in KNDy neurons, consequently influencing the pulsatile release of GnRH [67].

The surge mode of GnRH secretion occurs in adult females during the mid-cycle, wherein high concentrations of circulating estradiol, produced by the dominant ovarian follicles, stimulate GnRH neurons [68].

In animal models, this surge has been found to depend on the involvement of kisspeptin neurons in the AVPV in mediating the positive feedback of sex steroids on GnRH during the preovulatory period [69, 70]. In the presence of low serum estradiol and progesterone, Kisspeptin neurons located in the AVPV and activated, leading to an increased production of kisspeptin [71, 72].

In humans, there is no direct homologous area corresponding to the AVPV in rodents. Instead, Kp neurons, predominantly located in the infundibular nucleus and the POA of the hypothalamus, are likely responsible for the positive feedback of sex steroids, as previously suggested [73] (Fig. 1).

Kp acts as a ligand to the G protein-coupled receptor 54 (GPR54 or KISS1R), which is highly expressed in the hypothalamus, midbrain, pons, medulla, thalamus, hippocampus, amygdala, cortex, frontal cortex, striatum and also in the pituitary, placenta, pancreas, and spinal cord [74]. Lower concentrations of expression can also be found in the heart, muscle, kidney, liver, intestine, thymus, lungs and testis [75]. Kp acts directly on GnRH neurons (via GPR54) to modulate the pulsatile release of GnRH [76].

During a physiological ovarian cycle, FSH rises during transition from the luteal to follicular phase, and is necessary for follicular recruitment and growth. This rise in FSH and subsequent increase in Kp is directly linked to activation of KNDy neurons triggered by low serum estradiol and progesterone. Positive feedback is the driving force behind this process. Kp increases the amplitude of GnRH pulses while DYN simultaneously decreases GnRH pulse frequency resulting in the promotion of FSH release and overall increasing its serum concentration.

As ovarian follicles grow under the influence of FSH, E2 levels increase and downregulate KNDy secretion of Kp, NKB, and DYN. The subsequent reduction of GnRH amplitude with simultaneous increase in pulsatility leads to a reduction in gonadotrophin levels. The follicle with the largest number of FSH receptors continues its development as dominant follicle leading to a progressive increase of E2 [77].

High levels of estrogens restore and upregulate kisspeptin expression in the neurons of the AVPV and induce high amplitude GnRH pulses. At the same time, decreased dynorphin secretion from KNDy neurons in the ARC also promote an increase in GnRH pulse frequency leading to the preovulatory LH surge.

Neurons of the AVPV also receive afferent fibers from the suprachiasmatic nucleus, the neuronal origin of the circadian clock, which coordinates and provides precise timing for the LH surge. This surge leads to rupture of the dominant follicle, releasing the secondary oocyte and stimulating transformation of the remaining follicle structure (granulosa and theca interna cells) to form the corpus luteum which produces progesterone among other sex hormones.

The internalization of GPR54 from the surface of GnRH neurons after kisspeptin mediated activation terminates the LH surge. LH levels slowly decrease as a result of decreasing GnRH amplitude.

This decrease in LH plasma levels is responsible for involution of the corpus luteum followed by menstruation and subsequent luteal–follicular transition if the egg was not fertilized [78, 79].

## Kisspeptin and pubertal development

Kp plays an crucial role during puberty by stimulating GnRH secretion which in turn induces pituitary gonadotropin secretion [80]. Sexual immaturity observed in mice and humans resulting from the inactivation of GPR54 underscores the importance of Kp in the initiation of puberty [81]. It is noteworthy that mutations in genes such as KISS1, KISS1R and chromodomain DNA-binding helicase protein (CHD7), the latter of which is involved in the control of GnRH neurons, are associated with Congenital hypogonadotropic hypogonadism (CHH). CHH is a rare condition characterized by low serum levels of sex steroid hormones alongside low or normal plasma levels of gonadotropins. Typically, the remaining hypothalamic–pituitary function is unaffected, and the condition manifests as delayed pubertal development [82].

Furthermore, in 2008 Teles, M.G. et al. discovered a novel activating mutation of KISS1R in a girl with central precocious puberty, further emphasizing the significance of this neuropeptide in the physiology and pathology of puberty [83].

Metabolic status also plays a significant role in pubertal development. Adipose tissue is a primary source of leptin, which increases with the onset of puberty and subsequently triggers the release of kisspeptin from KNDy neurons [84]. Pubertal maturation coincides with a physiological increase in insulin resistance, suggesting that insulin may contribute body mass increase and overall organ development in ways that have previously been underestimated [85, 86].

## Kisspeptin and the metabolic control of reproduction

Precision in the regulation of energy balance is important for maintaining fertility. A negative energy balance (when energy expenditure outpaces fuel intake) in metabolism is known to promote signaling which inhibits the reproductive axis. Kisspeptin neurons are the link between energy status and fertility and have been studied in numerous animal models. It has been demonstrated that negative energy balance reduces endogenous expression of KiSS-1 [87, 88]. These findings have raised further questions as to the specific metabolic signals which drive this process.

**Fig. 2:**
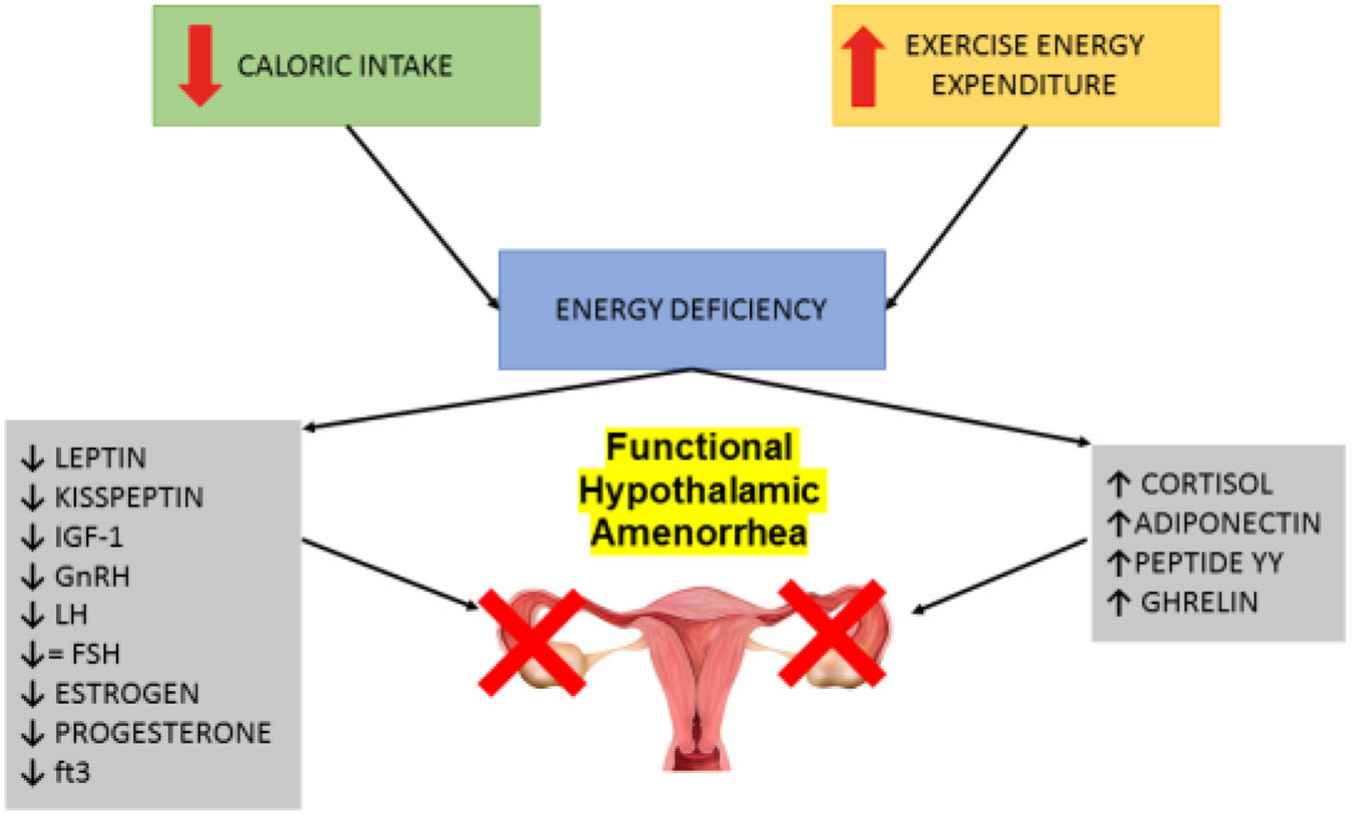
Many signals affect kisspeptin secretion, both positively and negatively. In cases of negative energy balance (either due to excess energy expenditure and/or deficit in caloric intake) the energy discrepancy activates a preservation mechanism halt reproductive function. This intervention is achieved by modulating the production of factors which affect hypothalamic GnRH secreting neurons and thus blocking reproductive ability through the reduction of LH secretion

It is generally hypothesized that a combination of various signals may result in regulation of kisspeptin secretion and subsequent fertility, thus supporting the important role of peptide molecules such as leptin, insulin, and ghrelin. Hunger hormones may serve as the critical link between the altered energetic balance which occurs in eating disorders and the development of functional hypothalamic amenorrhea. Leptin secretion is proportional to corporal fat stores and acts within the brain to signal adequate energy store and satiety [89]. In their pioneering study, Andrico et al. [90]. demonstrated that leptin levels in women with FHA were significantly decreased compared to controls. A chronic negative energy balance, or metabolic deficit will lead to chronic hypoleptinemia and to a compensatory downregulation of reproductive function [91]. In an animal model of food-restricted rats, administering leptin had a positive effect on infertility. Exogenous leptin was shown to promote ovulation and reverse the suppressive effect of energy deprivation on menstruation [92]. A subsequently conducted human treatment study, in which metreleptin was administered in women with FHA, yielded results paralleling those in the rat model with restoration of regular menses and an increase in estradiol and progesterone levels. Although adequate leptin concentration is essential for normal functioning of the reproductive axis, the neuroanatomical pathway linking leptin signaling to GnRH neurons is not yet fully understood as GnRH neurons do not possess a signaling isoform of the leptin receptor [93]. It has been suggested that leptin may stimulate KISS1 expression in AVPV neurons but these possible direct or indirect effects on ARC neurons continues to be heavily debated [93] (Fig. 2).

**Fig. 3:**
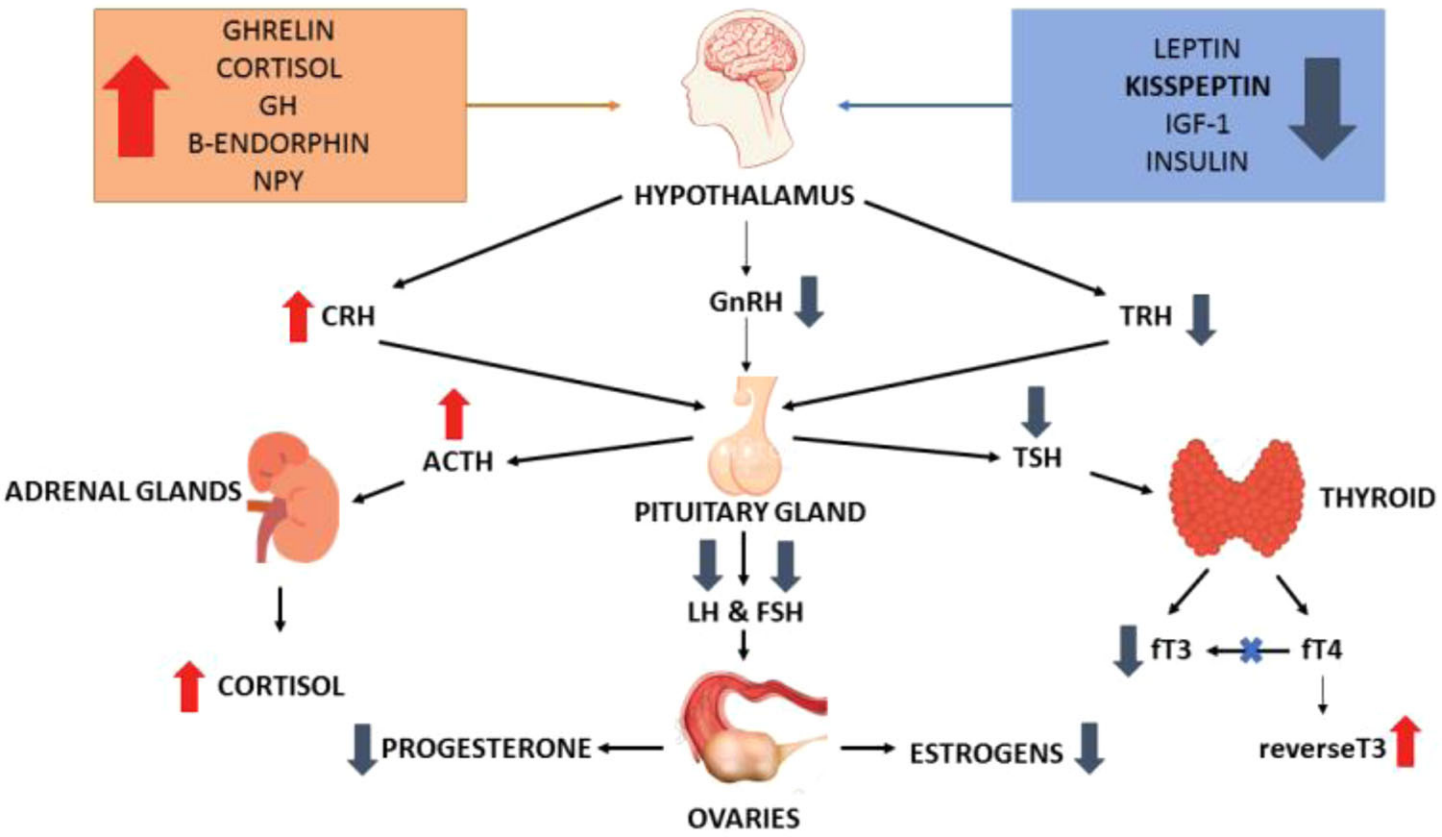
The complex endocrine and neuroendocrine impairment that occurs in functional hypothalamic amenorrhea (FHA). Non-exclusive peripheral metabolic signals trigger the specific neuroendocrine cascade causing FHA. Beyond direct metabolic signaling by leptin, Ghrelin, insulin, and cortisol on GnRH and kisspeptin producing neurons, reduction in the synthesis of fT3 also contributes to preservation of energy stores and reduction of heat production

Insulin is produced by pancreatic β-cells and is essential not only for the control of carbohydrate and fat metabolism but also acts directly on the hypothalamus regulating energy balance [94, 95]. A recent study involving food-restricted ewes has shown that the restoration of pulsatile LH secretion via return to normal feeding was preceded by an increase in circulating serum insulin and has backed the hypothesis that LH pulses are closely linked to energy availability and insulin [96] (Fig. 3).

Ghrelin is another promising candidate involved in metabolic signaling and in the central dysregulation of reproductive function in FHA. Ghrelin is an orthorexic peptide hormone secreted by specialized cells of the gastrointestinal tract and who’s secretion closely correlates with hunger. Although ghrelin acts on the same neurohormonal pathways as leptin, changes in ghrelin levels are closely tied to eating patterns and can be a marker of eating disorders. Schneider et al. [97] demonstrated that FHA is associated with elevated ghrelin and abnormal dietary behaviors. They also concluded that ghrelin likely contributes to delayed restoration of the menstrual cycle in women who are recovering from FHA. Intracerebroventricular administration of ghrelin in rats suppressed the pulsatile secretion of luteinizing hormone, providing further supporting evidence linking ghrelin to suppression of a normal menstrual cycle [98]. The precise mechanisms through which central ghrelin inhibits the reproductive system, however, remains unclear although it is believed that kisspeptin neurons in the hypothalamus may be central to this process.

Neuropeptide Y is one of the most potent orexigenic peptides identified in the brain to date. It has additional pleiotropic effects on metabolism, immune function, and reproduction. NPY has been shown to exert stimulatory effects on reproduction at multiple levels of the hypothalamic-pituitary-gonadal axis. Studies have shown that NPY is directly associated with gonadotropin release. NPY promotes secretion of luteinizing hormone releasing hormone (LHRH) and the resulting LH surge is correlated with increases in NPY levels [99]. Corio et al. [100] investigated NPY levels in female athletes with and without menstrual cyclicity. They found that NPY levels were significantly higher in subjects with normal cycling than in amenorrheic athletes, a finding which hints at a possible protective role of NPY in the maintenance of menstrual cycles. A complementary study by Meczeklaski et al., in which NPY levels were studied in patients with weight loss-related amenorrhea, found that basal serum NPY levels were lower in amenorrheic patients compared to menstruating women [101].

Recent evidence has suggested that together, leptin, insulin, and ghrelin modulate Kisspeptin producing neurons indirectly through the action of neuropeptide Y (NPY) and agouti-related peptide (AgRP) neurons as well as pro-opiomelanocortin (POMC)/cocaine and amphetamine-regulated transcript (CART) neurons located in the ARC [102]. Kisspeptin appears to be an essential link between energy balance and reproductive function.

It is becoming increasingly apparent that hunger hormones provide an additional level of communication between adipose tissue, the state of energy balance, and the functional integrity of the reproductive axis in humans.

## Functional Hypothalamic Amenorrhea as a form of stress induced reproductive impairment

Functional hypothalamic amenorrhea is a multifactorial condition characterized primarily by a decrease in hypothalamic gonadotropin-releasing hormone and chronic anovulation [103]. Sustained energy deficiency, such as is seen in participants of elite sports, excessive exercise, or in women with eating disorders can lead to reproductive dysfunction. This responsive dysfunction is considered a physiological safeguard to conserve energy during a sustained state of starvation by suspending the function of an energy-intensive process considered least essential to survival [104].

The pulsatile pattern of LH observed in patients with FHA is often that of either low amplitude pulses or night-time erratic pulses with an increased pulse amplitude resembling an early pubertal pattern [105–107].

As a consequence, ovarian function is impaired as estrogen production is reduced and ovulation does not occur. Progesterone is also very low or absent as it is produced primarily by the corpus luteum following luteinization of granulosa cells of the ovulating follicle. The menstrual cycle progressively becomes more irregular, deregulated, and over time is completely absent [28]. Nevertheless, in patients exhibiting FHA, initial follicle recruitment is preserved and AMH levels remain similar to those observed in healthy women [108].

FHA is frequently associated with endocrine impairments involving the thyroid and/or adrenal glands. However, it is also characterized by neuroendocrine dysfunctions, as evidenced by altered synthesis and release of several neurotransmitters, including serotonin, acetylcholine, dopamine and norepinephrine [55, 109, 110].

As a consequence of energy deficiency, either as suboptimal nourishment or excessive energy expenditure, a cascade of energy-conservation mechanisms is unleashed which include the reduction of leptin, glucose, insulin, insulin-like growth factor (IGF-1), kisspeptin (Kp), and elevation of ghrelin, growth hormone (GH), neuropeptide Y, and beta-endorphin [111–114].

Patients with FHA are typically characterized by lower serum kisspeptin and high serum CRH when compared to healthy individuals [115]. CRH levels in the elevated range are often found in patients with stress-related amenorrhea where it can directly inhibit pulsatile secretion of GnRH. Recent studies have found glucocorticoid and CRH receptors on KNDy neurons, suggesting that these neurons may play a bridging role between the hypothalamic-pituitary-adrenal (HPA) and the hypothalamic-pituitary-gonadal (HPG) axes [116, 117]. These findings link stress induced hormonal changes with the impairment of fertility in patients such as those with FHA.

A correlation is observed between serum Kp and LH secretion both in eumenorrheic healthy women and in patients with FHA [118–120]. Both hormones were found to exhibit a pulsatile pattern of release with corresponding peaks [118]. These findings demonstrate that the reproductive axis, even under pathophysiological conditions such as FHA, relies on kisspeptin to drive release of GnRH. Temporal coupling between kisspeptin and LH secretion is shown to be preserved in patients with FHA as well as in eumenorrheic patients with PCOS who have normal metabolic indices such as leptin and insulin. This Kp-LH coupling, however, disappears in oligomenorrheic patients with PCOS in whom higher levels of both insulin and leptin are observed [106, 107]. It is believed that metabolic signals may be responsible for this interference directly and/or indirectly.

A negative correlation has been observed between peripheral Kp levels and body mass index (BMI) as well as physical activity. This observation may imply that Kp reduction could act as a compensatory mechanism in anorectic individuals to prevent excessive physical activity and extreme BMI reduction [121, 122].

Ghrelin and IGF-1 have also been found to act as messengers between kisspeptin and the metabolic system. IGF-1 has been shown to stimulate KiSS-1 mRNA expression within AVPV neurons in female rats. This is in contrast to Ghrelin which acts as a suppressor of hypothalamic expression of KiSS-1 mRNA [123, 124] (Fig. 3).

FHA is often referred to as a proverbial “perfect storm” of endocrine and neuroendocrine impairment as many other pituitary hormones are independently affected by stress triggers. For example, TSH decreases under the influence of stress, as do fT4 and fT3 concentrations. Conversion of fT4 to its active metabolite fT3 is impaired and is instead converted to a biologically inactive reverse-T3.

In response to chronic stress, the hypothalamic pituitary adrenal axis is activated resulting in chronically elevated cortisol levels. After a temporaneous rise, plasma prolactin levels in women with FHA who endure chronic stress have been shown to drop. Pituitary PRL release in these patients also has a higher pulse frequency and lower pulse amplitude compared to healthy women [125].

Plasma levels of Brain-derived neurotrophic factor (BDNF), which plays an important role in the growth, development, maintenance, and function of several neuronal systems, is reduced in patients with FHA. The exact role of BDNF in the pathogenesis of FHA, however, is not yet fully understood [126].

The role of Kisspeptin is central to the regulation of GnRH secretion, its importance is evidenced by the close interaction it maintains with metabolic and psychological stress. The important response it demonstrates in FHA is a specific adaptive and defensive mechanism to avoid moving into a substantially more energy-inessive gestative state following ovulation. A recent study by Jayasena, C.N. et al. [119], in which a continuous IV infusion of kisspeptin-54 was administered in women with FHA, has demonstrated the ability to temporarily increase LH pulsatility. This work has pointed the way for potential kisspeptin-based therapies for use in women with secondary amenorrhea, a powerful tool to be used alongside rectification of metabolic impairment and/or chronic stressors in these patients [119].

## Neuroendocrine imbalances in FHA: norepinephrine, GABA, allopregnanolone, neuropeptide Y, melatonin, beta-endorphin and others

Functional hypothalamic amenorrhea has three primary causes, each being associated with an energy deficient state. Reduced energy intake, excess physical exercise, and stress are known to propagate neuroendocrine disturbances by acting on the pulsatile release of Gonadotropin-releasing hormone [127]. Oftentimes the causal driver leading to functional hypothalamic amenorrhea is a combination of all three factors [55]. As any of these coinciding conditions may lead to the development or further progression of FHA, a comprehensive approach needs to be used in its assessment. Numerous neuroendocrine regulating factors need to be considered when discussing the possible clinical manifestations and prognoses of FHA. There is a well-established link in women between stress level and the incidence of FHA. Neuroendocrine factors which are involved in regulating the hypothalamic-pituitary-adrenal axis (CRH, ACTH, and cortisol all of which dictate the severity of stress reactions) therefore have significant potential in affecting the incidence and evolution of FHA in women [128].

Neuropeptide Y, a neuroendocrine transmitter with widespread systemic functionality plays a central role in managing physiological stress reactions. NPY stimulates the release of GnRH [129], it also down-regulates corticotropin-releasing hormone and reduces circulating cortisol levels. This, in turn, has an anxiolytic effect. This interaction is fully reciprocal in that CRH has the ability to suppress the release of NPY [130].

Melatonin primarily regulates circadian rhythm but also has a functional influence on the HPG axis and modulation of stress reaction. Disturbance in sleep patterns - which can be both a result of, or result in, a decrease in melatonin levels - have been shown to lead to increased secretion of corticosteroids [131]. A decrease in melatonin levels can not only promote activation of the HPA axis but also negatively affect ovarian function. This association is not entirely surprising, seeing as high concentrations of melatonin have been found locally in follicular fluid of the ovaries [132].

Allopregnanolone is another regulator of stress alleviation. It is a natural product of progesterone metabolism and modulates the function of γ-Aminobutyric acid type A (GABA_A_) receptor by allosteric binding [133].

Gamma-aminobutyric acid (GABA) functions as the primary inhibitory neurotransmitter in the human brain [134]. GABAergic pathways, alongside input from glutamatergic neurons is involved in GnRH regulation via activation of KND neurons [28]. Allopregnanolone also has the ability to down-regulate activity in the HPA axis and in so doing reduces the severity of a stress reaction. It has been shown to provide a neuroprotective effect in conditions of prolonged stress and anxiety such as major depressive disorder or postpartum depression [133, 135].

In a similar fashion, beta-endorphins act as peptide agonists of the opioid receptor, are secreted systemically and found to ameliorate stress reactions. They function at a higher level in restoring homeostasis following exposure to stressors. The analgesic and immunity-boosting properties of endorphins, however, have led to suggest that they are limited to playing an alleviatory role in situations of acute stress as opposed to chronic exposure to stressors [136].

In contrast to neurons of GABAergic pathways which lead to activation and pulsatile release of GnRH, norepinephrine expressing neurons have been found to inhibit its secretion [137].

Energy supply, and disturbances therein, play such a crucial role in the development and progression of FHA that many neurohormones which regulate food intake such as insulin, leptin, and ghrelin are also tied into modulating GnRH release [28].

## Stress as etiology to hypothalamic amenorrhea: the influences of CRH, the hypothalamic-pituitary-adrenal axis, beta-endorphin, and Gonadotropin-Inhibitory Hormone (RF amide related peptide)

Hungarian-Canadian endocrinologist Hans Selye was a pioneer in the study of the biological basis for human stress response [138]. His groundbreaking work was first published in the late 1930’s and the decade that followed welcomed numerous studies focused on the interplay of the stress response mechanism and its influence on reproduction.

Stress was found to influence a wide range of factors, including reproductive function. So strong is this effect, that stress-related FHA is regarded as the main type of functional hypothalamic amenorrhea. Stress is at present the most significant factor responsible for the impairment of reproductive function [139].

Hypothalamic amenorrhea, when stress related, is the product of a complex interplay between the hypothalamic-pituitary-adrenal axis and the hypothalamic-pituitary-gonadal axis [28], with corticotropin releasing hormone as the common factor. In situations of stress, increased levels of CRH leads to the direct inhibition of pulsatile GnRH secretion, which further causes inhibition of the hypothalamic-pituitary-gonadal axis [140]. Additionally, when under stress CRH stimulates the release of beta-endorphin which in turn also impairs the pulsatile release of GnRH. Hypercortisolemia is a direct consequence of stress-induced CRH release. Increased levels of cortisol have been shown to exert an inhibitory effect on the release of GnRH and gonadotropin [141]. Administering CRH in healthy female volunteers was shown to inhibit pulsatile GnRH release [142]. On the other hand, administering a CRH antagonist stimulated the same pulsatile GnRH secretion [143].

A study by Podfigurna et al. reported that patients with FHA had lower serum kisspeptin levels and higher serum CRH levels when compared to healthy controls [144].

Meczekalski et al. [145] studied patients with stress-related FHA with normal body weight. They evaluated the response of ACTH, allopregnanolone, and cortisol to a CRH challenge in both patients with FHA and in healthy women. It was found that beyond high serum ACTH and cortisol levels, women with stress-related FHA also had low allopregnanolone levels at baseline. This was determined to be a result of impairment in both adrenal and ovarian synthesis. The blunted response of ACTH, allopregnanolone, and cortisol to CRH indicates a reduced sensitivity and expression of CRH receptors in hypothalamic amenorrhea.

Kisspeptin is a relatively novel factor found acting between the hypothalamic-pituitary-adrenal axis and hypothalamic-pituitary-gonadal axis. It is involved in the interplay between CRH and GnRH [146]. Increased CRH (when in stressful conditions) can directly inhibit GnRH secretion. Recent studies have revealed that CRH receptors and glucocorticoid receptors (both of which are involved in the stress response) are not expressed by GnRH neurons but rather are expressed by kisspeptin neurons. CRH receptors are expressed in both the anteroventral periventricular nucleus and the arcuate kisspeptin neurons. Glucocorticoid receptors are expressed only in AVPV kisspeptin neurons but not in ARC kisspeptin neurons [146].

These findings suggest that kisspeptin neurons may play a role as key communicators between the HPA axis (as a stress response system) and HPG axis (reproductive regulatory system). Beyond their direct action, kisspeptin neurons have been found to mediate the inhibition of reproductive function by hyperprolactinemia as well [146]. These mechanisms seem to be even more complicated initially thought. Gonadotropin-inhibitory hormone (GnIH), which in mammals is also called RFamide-related peptide 3 (RFRP), is a potent inhibitory regulator of gonadotropin-releasing hormone (GnRH) and subsequently of gonadotropins [147]. Recent evidence suggests that RFRP is involved in stress-induced reproductive dysfunction [148]. Increased cortisol can stimulate RFRP activity which in turn inhibits GnRH/LH secretion. Kisspeptin is also involved in this cascade, where it acts upstream to RFRP [148]. The decrease in kisspeptin which is seen when under the influence of stress leads to an increase in RFRP and subsequent activation of the HPA axis, this in turn leads to inhibition of the HPG axis [148]. It is becoming increasingly clear that kisspeptin and GnIH/RFRP together play a central role in stress-induced disruption of the HPG axis.

## Potential role of neuropeptides in the diagnosis and treatment of FHA (CRH, Kisspeptin, Leptin, NKB native hormones, or analogs use in treatment)

As FHA diagnosis in some patients is posing a challenge, neuropepties may prove instrumental in our future approach managing patients with FHA. FHA women are characterized with lower serum leptin concentrations, elevated ghrelin levels, high NPY levels and high-frequency LH pulses. Conceivably some of the neuropeptides described above will play a significant role in FHA diagnosis.

The recommended first line treatment for FHA is lifestyle modification. This involves the elimination of causative factors responsible for FHA in order to restore the body’s physiological balances. When simple lifestyle interventions prove ineffective, hormonal replacement therapy is implemented. This strategy, however, often does not adequately address issues of infertility, bone loss, and many other associated neuroendocrine abnormalities. It is this constellation of other abnormalities which form a starting point for investigating novel hormonal pathways as potential targets for treating FHA.

In a randomized trial where women with FHA were administered metreleptin, Chou et al. [149]. Demonstrated the potential for pursuing this line of investigation. They found that administering human recombinant leptin (metreleptin) in physiological doses over 36 weeks led to the restoration of menstruation, increased serum estradiol and progesterone levels, and a decreased serum cortisol level. Moreover, markers of bone resorption were also reduced, suggesting that continued use of metreleptin over a longer time frame may have a beneficial effect on bone metabolism. Complementary studies later confirmed the positive influence of leptin therapy on skeletal health by showing an increase in osteoblast proliferation and a decrease in osteoclastogenesis during treatment [150]. Correlative results were reported by Welt et al. [151] who observed that leptin administration resulted in follicular growth and ovulation as well as significantly increased levels of LH, estradiol, IGF-1, thyroid hormone, and bone-formation markers. These findings suggest that administering leptin in replacement doses may be a safe and effective therapy for women with FHA and can help shorten the time to recovery in this group over the use of lifestyle modification alone.

In 2009, Jayasena et al. [152] reported on their groundbreaking human trial in which kisspeptin injections were used for the first time in women with hypothalamic amenorrhea. They concluded that acute administration of kisspeptin-54 increased serum gonadotropin levels but repeat injections lead to a desensitization effect. Despite observing instances of tachyphylaxis during chronic administration, this study provided a basis for the potential use of kisspeptin therapy in the treatment of FHA. In the years following their initial work, subsequent studies were led by Jayasena et al. to establish an effective protocol for kisspeptin treatment. During this endeavor it was demonstrated for the first time that treatment with kisspeptin-54 restores basal and pulsatile LH secretion in women with HA [153].

A second major turning point in the study of kisspeptin was a report published by Abbara et al. [154] in which the effects of a KISS1R agonist MVT-602 was studied in healthy women and in women suffering from reproductive disorders. The authors compared the action of MVT-602 with native kisspeptin-54 observing a more rapid increase in serum LH level after administration of the KissR agonist in women with FHA than it did in healthy women and women with PCOS. Additionally MVT-602 was shown to have a greater potency and longer duration of action than did native kisspeptin-54. It was concluded that the KissR would have significant potential as a future alternative kisspeptin treatment. Despite promising data, the practicality of kisspeptin treatment in women with FHA remains a significant challenge for the future.

NKB plays a crucial role in regulation of the reproductive axis as complex relationships between ovarian sex steroids and neurokinin B were confirmed. Studies have shown the importance of the NKB system in modulating kisspeptin and GnRH secretion [155]. More recently, Matsuzaki et al. investigated NK3R agonist usage and concluded that stimulation of NKB signaling could recover suppressed LH secretion under acute fasting condition in male rodents [156]. As hypothalamic amenorrhea is associated with low GnRH pulse frequency and a subsequent decrease in LH production, restoring baseline LH secretion may potentially be achieved by treating with NKB agonists. These results are promising under the aspect of future NKB agonist application in FHA therapy.

In many cases, reproductive dysfunction is induced by everyday life stress and subsequent activation of the hypothalamic-pituitary-adrenal axis. As such, studies have postulated that CRH, the principal regulator of the HPA axis, could be leveraged as a target for innovative therapies. Matsuwaki et al. [157] have studied the relationship between HA and CRH. Using melanocortin-2 receptor deficient mice, the authors demonstrated that not only glucocorticoids, but also CRH itself is capable of disrupting estrous cyclicity. Moreover, they demonstrated that treatment using a CRHR1 antagonist restored the suppression of pulsatile LH secretion which was initially caused by acute stress. In a similar animal study using female macaques, treatment using a CRH-1 antagonist (antalarmin) was shown to restore LH pulse frequency following stress exposure. Further studies are still required, however, in order to establish the full effect these receptor antagonists have on the HPG axis [158]. These studies have established the groundwork for the development of future antiamenorrheic therapy.

## Research gaps and directions for the future

Despite the multi-directional approach to FHA pathophysiology, diagnosis and treatment there are still many areas not covered by previous research. Better understanding of underlying neurohormonal mechanisms may allow the creation of markers that will facilitate FHA diagnosis. Low BMI, estradiol, LH and LH:FSH ratio are well known FHA characteristics that distinguish women with FHA for overall population yet many patients still experience delays in the diagnosis. Creation of an extended neurohormonal profile, including decreased serum NKB concentrations, reduced kisspeptin levels, decreased leptin, elevated ghrelin and decreased NPY, may not only improve FHA detection but also enable to isolate group of women in high risk of hypothalamic amenorrhea development which require greater attention. Moreover, as mentioned above, these neurohormones can constitute potential new therapeutic targets. Additional studies with larger sample sizes are needed to determine clinical utility of metreleptin, kisspeptin, NKB agonists and CRHR1 antagonist in novel, targeted treatment of functional hypothalamic amenorrhea. Initial studies appear to be promising, although further research is needed before they can be fully incorporated in clinical practice.

## Conclusions

Functional hypothalamic amenorrhea is the most common cause of secondary amenorrhea in women of reproductive age and as such its clinical significance cannot be overstated. Although we understand FHA to be the result of impaired GnRH secretion, the underlying pathophysiology is very complex and remains only partially understood. We know that numerous neuropeptids, neurosteroids, and neurotransmitters are involved in the progression of FHA, and work continues to fully classify their function. With recent advances in identifying and describing the role of an ever-growing number of hypothalamic and extrahypothalamic substances, hope remains strong for their use in the diagnosis and treatment of FHA. Efficient and accurate diagnosis, treatment, and monitoring of FHA will help to protect young women from the most severe complications of hypoestrogenism.
