# Correction: Neuroendocrine disturbances in women with functional hypothalamic amenorrhea: an update and future directions

**DOI:** 10.1007/s12020-024-03713-7

**Published:** 2024-02-17

**Authors:** Błażej Męczekalski, Olga Niwczyk, Christian Battipaglia, Libera Troia, Anna Kostrzak, Gregory Bala, Marzena Maciejewska-Jeske, Alessandro D. Genazzani, Stefano Luisi

**Affiliations:** 1 Department of Gynecological Endocrinology, Poznan University of Medical Sciences, Poznan, Poland; 2 Gynecological Endocrinology Center, Department of Obstetrics and Gynecology, University of Modena and Reggio Emilia, Modena, Italy; 3 Department of Gynecology and Obstetrics, Maggiore della Carità Hospital, University of Eastern Piedmont, Novara, Italy; 4 UCD School of Medicine University College Dublin, D04 V1W8 Dublin, Ireland; 5 Department of Molecular and Developmental Medicine, University of Siena, Siena, Italy

Correction to: Endocrine (2023)

10.1007/s12020-023-03619-w published online 07 December 2023

In the original publication of the article, all the author’s first names and family names have been published in reverse order.

The correct order should be as follows:

**Table:** 

Surname	Name
Meczekalski	Blazej
Niwczyk	Olga
Battipaglia	Christian
Troia	Libera
Kostrzak	Anna
Bala	Gregory
Maciejewska-Jeske	Marzena
Genazzani	Alessandro D.
Luisi	Stefano

This has been corrected in this paper.

